# Thermal and Viscoelastic Responses of Selected Lignocellulosic Wastes: Similarities and Differences

**DOI:** 10.3390/polym15092100

**Published:** 2023-04-28

**Authors:** Daniela Ionita, Mariana Cristea, Susana Felicia Cosmulescu, Georgeta Predeanu, Valeria Harabagiu, Petrisor Samoila

**Affiliations:** 1 “Petru Poni” Institute of Macromolecular Chemistry, Aleea Grigore Ghica Voda 41A, 700487 Iasi, Romania; ionita.daniela@icmpp.ro (D.I.); hvaleria@icmpp.ro (V.H.); samoila.petrisor@icmpp.ro (P.S.); 2 SC Cosfel Actual SRL, Calea Grivitei 95-97, 010705 Bucharest, Romania; cosfel@gmail.com; 3 Research Center for Environmental Protection and Ecofriendly Technologies, University POLITEHNICA of Bucharest, Strada Gheorghe Polizu 1-7, 011061 Bucharest, Romania; gpredeanu@gmail.com

**Keywords:** lignocellulosic biomass, thermogravimetric analysis, high resolution TGA, modulated TGA, dynamic mechanical analysis, differential scanning calorimetry

## Abstract

Woody lignocellulosic biomasses comprise the non-edible parts of fruit trees. In recent years, the exploitation of this biomass has been widening in order to mitigate environmental issues. At the same time, this waste could be transformed into a value-added product (active carbon by pyrolysis, isolation of nanocellulose, oils or proteins). For either valorization path, a complete thermo-mechanical characterization is required. A detailed thermo-mechanical study (TGA, DSC, DMA) was performed on two types of lignocellulosic wastes, with and without kernels: on one side, the walnut shells (WS) and the pistachio shells (PsS) and, in the second category, the apricot seeds (AS), the date seeds (DS), and the plum seeds (PS). The results of the sample-controlled thermal analyses (HiRes TGA) evidenced a better resolution of the degradation steps of WS. Kinetic studies conducted also by conventional TGA (Flynn–Wall–Ozawa) and modulated TGA (MTGA) allowed us to make comparative reasonings concerning the degradation of the investigated biomasses. The DMA results revealed the effect of water traces and oil kernels on relaxation and supported the atypical DSC endotherm emphasized in the freezing temperature domain.

## 1. Introduction

The survey of the current scientific literature dedicated to what scientists call biomass refinery reveals the astonishing interest in putting forward the potential of biomasses and the extraordinary progress made in recent years in the field. The circular economy strategy includes, among its targets, the valorization of agro-industrial waste, which represents a rich source of lignocellulosic biomass, principally composed of cellulose, hemicellulose, and lignin [1,2]. The woody biomass waste derived from fruit trees consists of non-edible products such as shells (pistachio, walnut) and seeds (olive, plum, peach, apricot, date, cherry) [3,4,5,6,7,8,9,10,11].

There is a tremendous flexibility in the exploitation of lignocellulosic biomass. Efforts have been made in the direction of the production of activated carbon (biochar and hydrochar) largely used for the adsorption of metals from aqueous solutions or dedicated to the fabrication of natural sanitizers [11,12,13,14,15,16,17]. The incorporation of biomass fillers into polymeric matrices to obtain biocomposites represents a trend in major significance [18,19,20,21,22,23,24]. Moreover, lignocellulosic biomass is a source of cellulose nanocrystals [25,26,27], oligosaccharides, lignin, and various ingredients for the cosmetic, pharmaceutical, and food industries (such as oils or proteins) [28,29,30,31,32,33].

It is well known from biomass waste practices and the literature that the structural (Fourier-transform infrared spectroscopy, Raman spectroscopy) [13,16,31,34,35,36,37], morphological (X-ray diffraction, scanning electron microscopy, atomic force microscopy) [31,34,36,38,39], mechanical [40,41], and thermal characterizations (thermogravimetric analysis—TGA; differential scanning calorimetry—DSC; dynamic mechanical analysis—DMA) [31,35,37,40,42,43,44,45] of these lignocellulosic compounds represent a key stage before addressing any application field. In this framework, a substantial body of research has been devoted to thermogravimetric analysis of biomass powders because it offers a deep understanding of the nature of pyrolysis phenomena and their kinetics. The results are indispensable for conceiving a strategy related to the biomass conversion application [46,47,48,49]. For example, the biomass intended for active carbon was investigated by TGA before and also after the adsorption process. State diagrams of foods have been constructed with the help of DSC results [50]. However, DSC investigations of biomass wastes are much less present in the published results than TGA, possibly due to the rigid structure of lignocellulose not producing measurable heat flow shifts during thermal scanning above room temperature [37]. Rahman et al. have published extensive DSC studies of pit powders, enlarging the investigated temperature range to negative temperatures [42]. The phenomena were commented on in terms of glass transition, ice formation, and fat melting. In the study of the molecular mobility of macromolecular compounds (natural or synthetic), in the situation of poor DSC signal, DMA can be the method of choice in identifying faint transitions, provided that the sample has a regular shape with measurable dimensions (films, bars, discs) [51]. Therefore, the sample can be fixed in an appropriate attachment and the proper mode of deformation can be applied: tension, bending, compression, or shear. A great variety of DMA studies have been performed for wood, especially to explore the influence of water on the viscoelastic properties. This is an easier task as compared to lignocellulosic wastes because wood fulfills the DMA requirements for these types of studies [52,53,54,55]. Furthermore, there are alternatives for performing viscoelastic measurements on biomass powders. One option is to use the material pocket for samples that are not self-supporting. Nonetheless, the results offer only a qualitative perspective [56]. Another alternative was employed by Guigo et al., who used solid pellets of isolated lignin powders for conducting rheometric measurements in the oscillating mode [57]. Very recently, Al-Khali et al. applied the same modality for monitoring the mechanical relaxation of de-fatted date-pits on powder tablets [58]. Despite the fact that both wood and woody biomass wastes are lignocellulosic biopolymers, the contents of hydrophilic components (cellulose, hemicellulose) are higher in the wood, which make it much more hygroscopic [23]. Additionally, the bulk microstructures of them could be different, as it has been described in walnut shells [59]. A distinct DMA behavior is expected for woody biomass as a consequence of its powdered form.

The paper reports further evidence of the thermo-mechanical behavior originating from TGA, DSC, and DMA experiments performed on two types of lignocellulosic wastes, with and without kernels. The first category comprises walnut shells (WS) and pistachio shells (PsS) that do not have kernels. The apricot seeds (AS), date seeds (DS), and plum seeds (PS) belong to the second category; they were grounded without removing kernels. All the TGA studies performed on lignocellulosic materials have described their degradation process as overlapping degradation steps of hemicellulose, cellulose, and lignin. In parallel with conventional TGA (constant heating rate in the investigated temperature interval), sample-controlled thermal analysis (HiRes TGA) [60,61] and modulated temperature TGA (MTGA) [62,63] will be conducted on WS in order to improve the resolution of overlapping processes. Specific for HiRes TGA is the use of the feedback from the sample to control the heating profile. In this way, the heating rate trends to zero when degradation occurs and has a value defined by the experiment in the absence of degradation. In MTGA, a modulated temperature profile superimposes the conventional one. The aim of the kinetic approach is to determine the activation energy, which will support the whole rationale of the TGA study. The investigations also project to probe the molecular mobility phenomena (transitions/relaxations), as they are evidenced by DSC and DMA measurements on the mentioned lignocellulosic wastes that were not examined thus far.

## 2. Materials and Methods

### 2.1. Materials

Walnut shells (WS), apricot seeds (AS), and plum seeds (PS) were obtained from local farmers. Date seeds (DS) and pistachio shells (PsS) were purchased from the local market. The contents of the lignocellulosic components, as the literature reports, are included in Table 1. Of course, the contents of cellulose, hemicellulose, and lignin may slightly differ depending on the origin of biomass.

All biomasses were conditioned as in our previous work [65,66]. Briefly, they were primarily washed with distilled water to remove dust and other impurities, followed by drying in an oven at 105 °C for 24 h. Then, they were mechanically ground with a Knife Mill Pulverisette 11 (Fritsch, Idar-Oberstein, Germany). The resulting crumbs were sieved using a Retsch AB system (Germany), and the powder fraction with dimensions between 125 μm and 250 μm was retained (ISO 3310-1). It was kept at room temperature, in a dry place, until use.

### 2.2. Methods

#### 2.2.1. Thermogravimetric Analysis

The thermogravimetric analyses of the powdered samples were carried out on a Discovery TGA 5500 (TA Instruments, New Castle, DE, USA). The tests were conducted using three heating rate algorithms: constant heating rate, dynamic heating rate (HiRes TGA, and modulated approach (MTGA). The temperature increased from ambient temperature to 700 °C. The experiments were performed in a nitrogen atmosphere at a flow rate of 25 mL/min. Around 6 mg of the samples was evenly and loosely distributed on a platinum pan in order to ensure good temperature uniformity during the measurement.

In the case of conventional TGA, the samples were heated at four constant heating rates: 2 °C/min, 5 °C/min, 10 °C/min, and 20 °C/min.

The HiRes TGA experiments were performed at sensitivity 1 and resolution 6, using an initial heating rate of 20 °C/min.

A slow heating rate of 2 °C/min was used in MTGA to provide enough modulation cycles for each reaction step. A temperature modulation amplitude of ±5 °C and a period of 200 s were used.

The activation energy (E_a_) for biomass pyrolysis was estimated with the help of the Flynn–Wall–Ozawa isoconversional method (method free) and modulated TGA [67]. Under linear heating, E_a_ was calculated as the slope of the straight line determined by plotting the natural logarithm of the heating rate (ln β) vs. reciprocal temperature (1/T) at different degrees of conversion:

Ea=−Rb×dlogβd1T

where E_a_ is the apparent activation energy (J/mol), β is the heating rate (°C/min), R is the gas constant (8.314 J/mol·K), T is the temperature at a specific conversion (K), and b is a constant (0.457).

In MTGA, the E_a_ for the conversion of interest was obtained in a single experimental run.

#### 2.2.2. Differential Scanning Calorimetry

Differential scanning calorimetry (DSC) was performed using a Discovery DSC 250 (TA Instruments, New Castle, DE, USA) under a nitrogen atmosphere (50 mL/min). The sample (powder) with a mass of 6 mg was sealed in an aluminum crucible. A heating–cooling–heating program with a heating rate of 20 °C/min was employed between −100 °C and 200 °C.

#### 2.2.3. Dynamic Mechanical Analysis

Dynamic mechanical analysis (DMA) tests were carried out on an RSA G2 analyzer (TA Instruments, New Castle, DE, USA) in compression mode. The disks for the experiments, having 15 mm diameters, were obtained using a Specac Atlas Manual Hydraulic Press. The disks were deformed with a 0.03% strain, which was well within the viscoelastic linear range. The changes in the storage modulus (E′), loss modulus (E″), and loss factor (tan δ) were recorded as a function of temperature and frequency.

The dimensional stability of the disks was tested in the frequency range of 0.01 ÷ 100 Hz and in time (150 min).

The isochronal experiments (1 Hz) were run with a heating rate of 2 °C/min from −100 °C up to 200 °C. Multifrequency experiments were performed at 0.1, 0.5, 1, 2, 5, and 10 Hz.

The heating (2 °C/min)–cooling (2 °C/min)–heating (2 °C/min) experiment was performed to prove the release of volatile compounds during the first heating step.

## 3. Results and Discussion

### 3.1. Thermogravimetric Analysis

**Figure 1:**
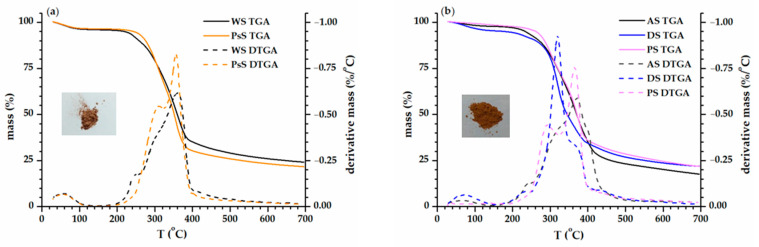
Conventional thermal degradation curves for WS and PsS (**a**) and AS, DS, and PS (**b**) biomasses.

First, the conventional TGA provided essential information required before performing any DSC or DMA investigations. For DSC cell safety reasons, the end temperature of any DSC experiment was established as a function of the thermal degradation onset. Additionally, the knowledge of the degradation steps was helpful for suitable ascertainment of the origin of phenomena featured in DMA. Figure 1a,b show the conventional TGA and temperature derivative (DTGA) curves of the two sets of biomass investigated. The main thermal characteristics associated with them are included in Table 2.

The evaporation of water traces and volatile compounds was responsible for the mass loss observed from room temperature to 150 °C. The most significant mass loss occurred within the temperature range of 200–450 °C (Figure 1a,b). The degradation pattern of the lignocellulosic biomass was largely discussed in the literature [19,46,47,68,69]. Previous reports have accounted for the complex degradation process that involved mainly three overlapping degradation steps: hemicellulose started first (200–250 °C), but its degradation ended after the pyrolysis onset of cellulose (around 350 °C). Lignin degraded over the entire mentioned temperature interval, continuing beyond 450 °C. In this sense, the mass loss curves had a gentle gradual decline until the end of the experiment (700 °C) (Figure 1). Investigations of related systems supported this trend even until 950 °C, where the residue weighed up to 20% [26].

According to the degradation temperatures (T_deg onset_, T_max_, and T_10_), the PS biomass was the most stable. However, the WS biomass had the lowest mass loss during the main degradation stage (200–450 °C) and the higher residual content at the end of the experiment (Table 2). It was not excluded that the robust intimate structures of the walnuts made it less liable to final degradation as compared to the other biomasses [59].

A closer look at the DTGA curves (Figure 1, dotted lines) revealed that WS and PsS presented two maximums, while three maximums were noticed for AS, DS, and PS. The first two peaks displayed in the main degradation stages could be associated with the degradation of cellulose and hemicellulose. Additionally, the occurrence of the third DTGA peak (around 450 °C) in the AS, DS, and PS biomasses could principally describe the degradation of the lignin. As mentioned before, the decomposition of lignin is not easily identified in a conventional TGA. A dynamic TGA investigation (HiRes TGA) can shed some light on this aspect.

**Figure 2:**
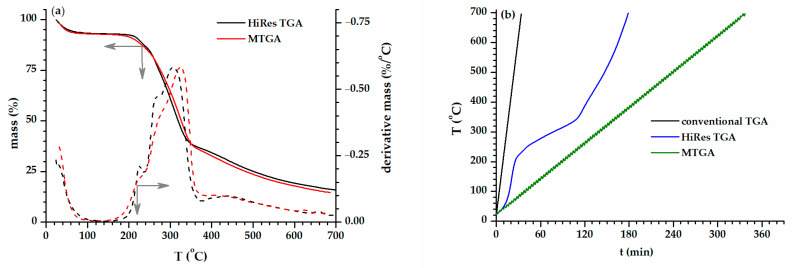
(**a**) Experimental HiRes TGA and MTGA curves obtained for the thermal degradation of WS. (**b**) Temperature profile vs. time for the three types of TGA: conventional, HiRes, modulated.

The results of the HiRes TGA and MTGA performed on the WS are displayed in Figure 2a. Figure 2b exhibits a graphic representation of the time–temperature profile for the three types of TGA experiments (conventional, sample-controlled, and modulated).

The main degradation peak of the WS shifted to lower values (308.4 °C, HiRes TGA; 324.8 °C, MTGA) as compared to the maximum obtained in conventional TGA (364.8 °C). However, only the HiRes TGA led to better resolutions of the thermal events in a reasonable time span: well-marked shoulders at 226.4 °C and 266.5 °C on derivative TGA and distinct degradation steps on TGA. The drop of the heating rate to a very low value (1.5 °C/min) during the mass loss periods could explain the better result obtained with the HiRes TGA. Additionally, a shoulder displayed around 450 °C corresponding to lignin was separated from the main peak. In a dynamic experiment, the time of investigation is longer than that of the conventional one (Figure 2b), and some details of the process could be better put in evidence.

**Figure 3:**
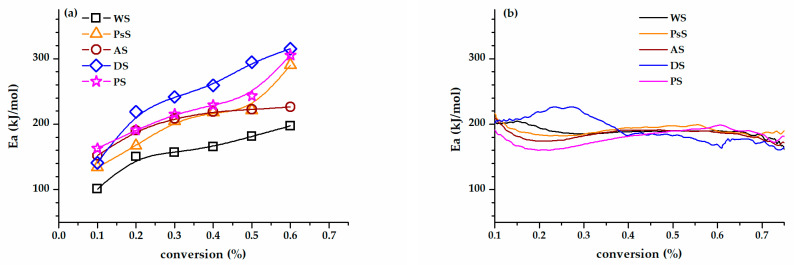
The variation in E_a_ for the biomasses investigated, as a function of the conversion degree, calculated with the Flynn–Wall–Ozawa equation (**a**) and obtained from the MTGA experiment (**b**).

For all types of biomasses studied, the values of E_a_ were calculated according to the Flynn–Wall–Ozawa equation at conversions between 0.1 and 0.6 (Figure 3a). All regression coefficients (R^2^) were greater than 0.97. It was evident that the values of E_a_ were not constant but had an increasing trend with the conversion. Torres-García et al. reported that the lignocellulosic systems of peanut shells displayed analogous behavior, i.e., the E_a_ values increased with conversion [70]. This fact confirmed the biomass complex multistep degradation mechanism, consisting of different concurrent and consecutive reactions [71]. Moreover, particular features should be underlined for the investigated biomasses. In the conversion interval of 0.1–0.5, the values of E_a_ corresponding to WS and PsS were lower than those of AS, DS, and PS.

The Flynn–Wall–Ozawa model free procedure has its limits. Performing the experiments at different heating rates means to shift the balance of concurrent/consecutive decomposition reactions of hemicellulose, cellulose, and lignin, which are defined by nonidentical mechanisms [72]. On the contrary, the MTGA approach provides the values of E_a_ for the whole range of the experimental conversion in a sole experiment in an incomparably less time than conventional TGA and with improved accuracy (Figure 3b). Accordingly, the variation in E_a_ can be divided into two zones. The first one, which corresponds to the conversion between 0.1 and 0.4, is characterized by values of E_a_ that decrease from 224 kJ/mol (DS) to 161 kJ/mol (PS). The convex shapes of the WS, PsS, AS, and PS curves suggest the association of the first stage with the end of hemicellulose degradation. The concave shape of the DS curve denotes that the whole range of hemicellulose degradation is captured in the first step. The second stage, which marks the beginning of the cellulose decomposition, is associated with small raise of E_a_ for the investigated biomasses (183–198 kJ/mol).

### 3.2. Differential Scanning Calorimetry

**Figure 4:**
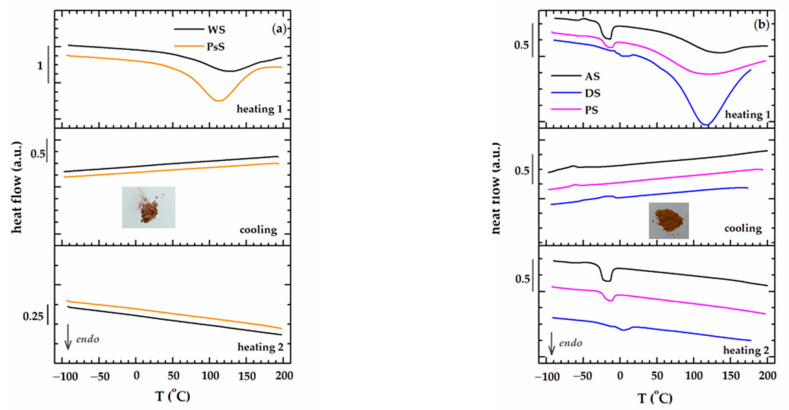
The result of DSC scans (heating 1–cooling–heating 2) for lignocellulosic biomass wastes: (**a**) WS and PsS; (**b**) AS, DS, and PS.

**Figure 5:**
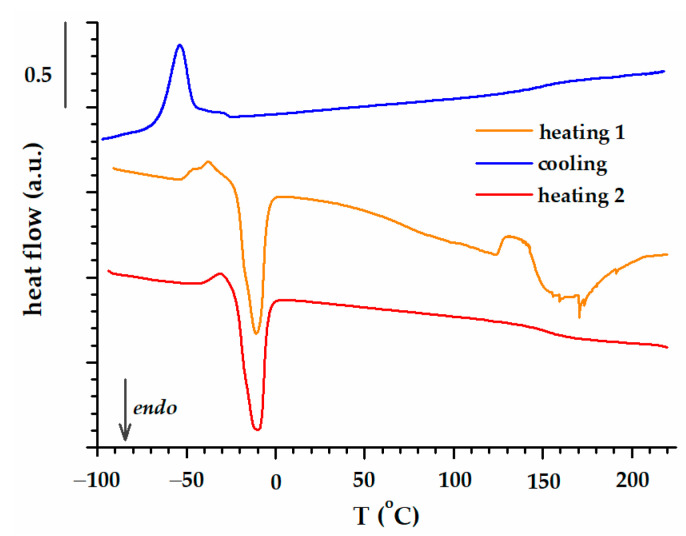
The DSC scan result for PS kernels.

Figure 4a,b illustrate the DSC curves (first heating–cooling–second heating) of the two types of biomass wastes used: WS and PsS on one side and AS, DS, and PS, on the other side. The very large endothermic peaks that appear in the high temperature region on the first heating stage are associated with the vaporization of water traces and other volatiles. The lignocellulosic biomasses without kernels (WS and PsS) do not display any other thermal events during cooling and the subsequent second thermal heating. The AS, DS, and PS biomasses evidently exhibit a secondary endothermic peak in both the first and the second heating steps, centered at −18 °C (AS), −13 °C (PS), and 5 °C (DS). The DSC experiment performed on the kernel of plum after removing the shell, in exactly the same conditions as for seed, displays evident endothermic peaks in the first and second heating steps and one exothermic peak during cooling (Figure 5).

Similar results were obtained previously by other research groups who made extensive investigations of date seeds [42]. They associated the phenomena occurring in the temperature range −25–0 °C with the presence of kernel oils (order–disorder transition).

### 3.3. Dynamic Mechanical Analysis

**Figure 6:**
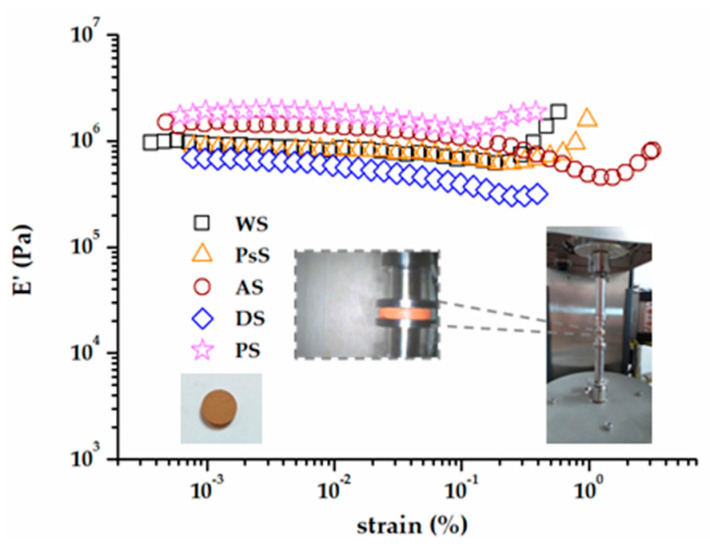
The dependence of the storage modulus (E′) on the applied stress at 20 °C and 1 Hz.

The viscoelastic properties of the biomass wastes were investigated in the oscillatory compression mode. This approach was performed to confirm the DSC results and also to achieve more insight into the phenomena occurring at a molecular level. The linear viscoelastic region (LVR) was determined to establish the strain range, where the results were not affected by the applied strain [73,74]. The storage modulus (E′) vs. the applied strain is plotted in Figure 6. Usually, the superior limit of LVR is marked by the decrease in E′ modulus. After this point, structural breakdown is possible, and the results of measurements become erroneous. A strain of 0.03% was chosen to keep the behaviors of all the biomass samples inside LVR.

However, the E′ modulus did not maintain a decreasing trend. At some point (strain values around 0.2%), an upward shift of E′ was noticed (Figure 6). This was an indication that the rigidity of the sample increased from this value of strain onward, most likely due to the cohesion between the tiny grains’ growth.

**Figure 7:**
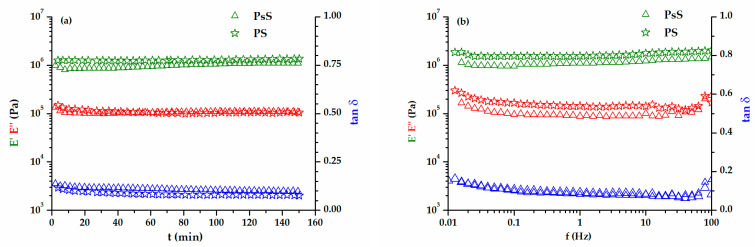
The dependence of the viscoelastic characteristics (E′, E″, and tan δ) on time (**a**) and frequency (**b**) at 20 °C, for PsS and PS biomasses.

The final aim of our DMA study was to determine the viscoelastic behavior in an isochronous temperature scanning DMA experiment. According to the onset temperatures of degradation (Table 2), the DMA experiment could be performed until 200 °C. Given the particular behavior of the biomass disks, it turned out that the stability of these disks should be checked by applying a 0.03% oscillation deformation for 150 min. This time interval represented the estimated duration of the isochronal DMA experiment conducted between −100 °C and 200 °C, with 2 °C/min. For the same reason, the variations in the viscoelastic properties of the biomass disks with frequencies were checked. This aspect could also be important for the transporting, storing, or handling of biomass powders. The variations in the viscoelastic parameters E′, E″, and tan δ with time and frequency were exemplified in Figure 7a,b for two types of biomass wastes (PsS and PS), without and with kernel. The disks were quite stable all over the measured intervals. Only a small increase in the loss modulus was noticed at frequencies higher than 50 Hz; the cohesiveness of the disks could have lessened at frequencies higher than 50 Hz.

**Figure 8:**
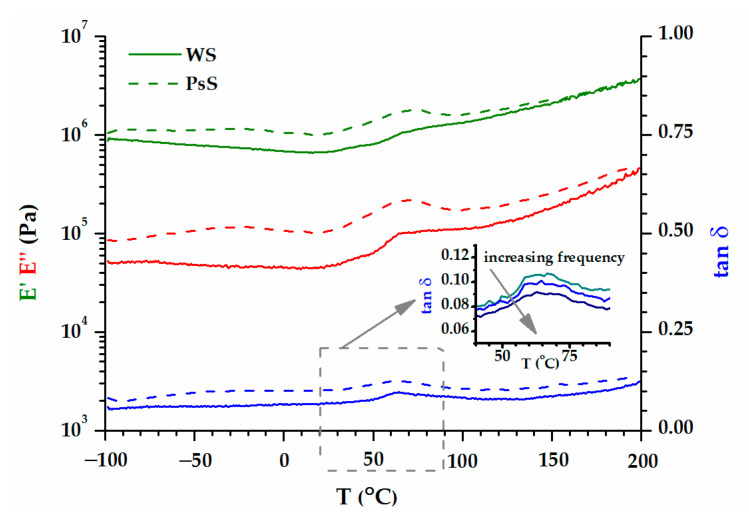
The variation in E′, E″, and tan δ with temperature, at 1 Hz, for WS and PsS biomasses. The inset includes the tan δ pattern associated with the increase in E′ modulus, resulting from a multifrequency experiment.

**Figure 9:**
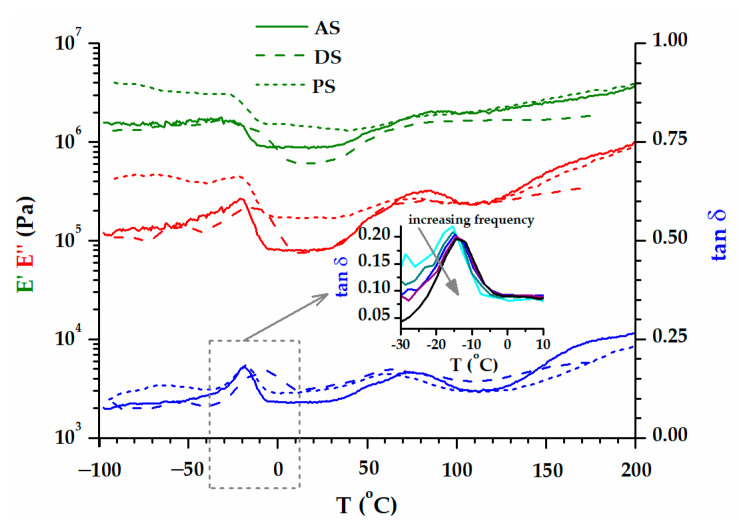
The variation in E′, E″, and tan δ with temperature, at 1 Hz, for AS, DS, and PS biomasses. The inset includes the multifrequency tan δ plots associated with the decrease in E′ modulus before 0 °C.

The dependence on viscoelastic behaviors with the temperatures of the two groups of biomass wastes is represented separately in Figure 8 (WS and PsS) and 9 (AS, DS, PS). The E′ modulus of the samples at room temperature was around 10^6^ Pa, a value that was confirmed by the previous time and frequency sweep tests (Figure 7). The elastic component was dominant all over the temperature range investigated. A preliminary examination of the results did not reveal a certain glass transition region. However, two phenomena were more prominent, which occurred in the temperature ranges −50–0 °C and 50–100 °C. In the first case, there was a decrease in the E′ modulus, which was more intense for the biomass powders that also contained kernels (Figure 9). In the second case, the E′ modulus rose as the temperature became higher. Both the decreases and the increases in the elastic responses had associated E″ and tan δ peaks. Evidently, the increase in E′ meant a gain in rigidity that was principally associated with the elimination of volatile compounds [75]. The origin of the large DSC endothermic peak that appeared in the first heating (Figure 4b) was reconfirmed. The main question was the origin of the events emphasized close to the freezing temperature, which will be discussed further down.

The issue with interpreting the DMA results is the fact that not any E″ or tan δ peaks could be associated with relaxation. Relaxation is the process of going from a state where the experimental frequency is too fast for a certain mode of molecular motion to a state where it is too slow [76]. Practically, the evolution of the tan δ peak in the multifrequency experiment confirms whether a tan δ peak is evidence for the relaxation process [77,78]. The inset of Figure 8 shows that the tan δ peak associated with loss of volatile compounds (E′ raise) does not depend on the frequency, and it is not a relaxation. However, the frequency dependence of the first tan δ peak has the characteristics of relaxation (inset of Figure 9). It turns out that the presence of oil in the powders (AS, DS and PS) generates relaxation due to the order–disorder transition. The viscoelastic behaviors of WS and PsS (Figure 8) are devoid of this feature.

**Figure 10:**
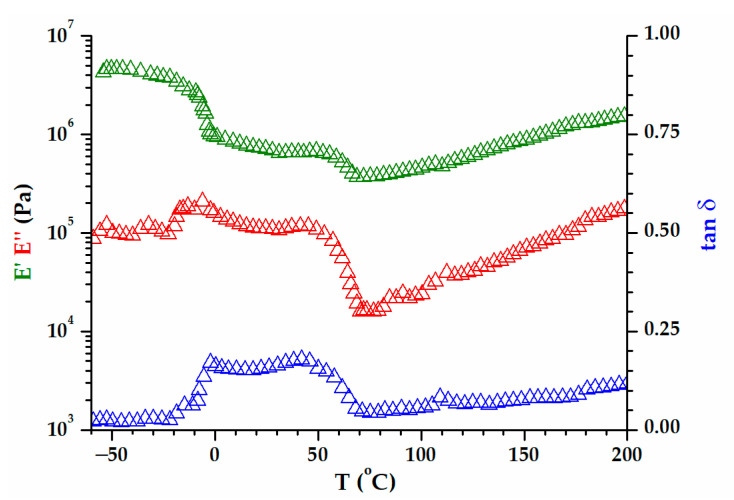
The variation in E′, E″, and tan δ with temperature, at 1 Hz, for wet PsS.

The difficulties in the pyrolysis of biomass wastes stem from the presence of water traces. It was reported that the water content in a biomass suitable for pyrolysis should not be higher than 30% [79]. The DMA behavior is very sensitive to the presence of water associated with the polymer chains [78,80,81]. For example, a disk of PsS was maintained in a humid atmosphere, and the absorption of water was very evident. The DMA behavior of this disk is represented in Figure 10.

A succession of overlapping relaxations was detected due to the association of water with the polymeric biomasses. The antiplasticizing effect of water, defined by the increase in the E′ modulus of a polymer in the presence of water, is a phenomenon characteristic to biopolymers. It had been recounted and put in evidence previously by DMA [82,83]. The viscoelastic behavior of wet PsS reflected a broadening of the relaxations and their evident shifts to higher temperatures when compared to the dry PsS (Figure 8). Moreover, the E′ modulus at −50 °C increased considerably as a result of the increased rigidity in the presence of water (dry PsS: 10^6^ Pa; wet PsS: 4.5 × 10^6^ Pa).

## 4. Conclusions

Thermo-mechanical characteristics are very important for the controlled pyrolysis of biomass wastes. Advanced thermogravimetric techniques (HiRes TGA and modulated TGA) were applied to a series of woody biomass wastes, shells, and seeds with kernels (WS, PsS and AS, DS, PS). HiRes TGA proved useful to better separate the degradation stages of lignocellulose originating from walnuts. The presence of kernels in biomass wastes was found to increase the E_a_ of thermal degradation, calculated using the Flynn–Wall–Ozawa method. This study furthered the understanding of the complex mechanisms of lignocellulose thermal degradations resulting from MTGA, which provides E_a_ values in a single experiment at each conversion degree. The large DSC endothermic peak and the increasing trend in storage modulus resulting from the DMA, exhibited after 50 °C, confirmed the elimination of volatiles. The presence of the complex DSC endothermic and tan δ peaks (DMA) with relaxation features, in the freezing temperature range, supported the occurrence of the order–disorder transition in the seeds with kernels. The traces of water could have antiplasticizing effects.

## Figures and Tables

**Table 1:** The contents of the main components of biomasses, as is reported in the literature.

	Hemicellulose	Cellulose	Lignin	Reference
WS	22.40	23.90	50.30	[4,19,22]
PsS	31.40	38.10	25.60	[26]
AS	17.01	29.57	47.97	[22,24]
DS	26.80	23.90	21.60	[14]
PS	24.46	21.34	42.15	[64]

**Table 2:** The main thermal degradation characteristics of the investigated biomasses, resulting from conventional TGA.

Sample	Main Degradation			T_10_^(3)^ (°C)	Residue ^(4)^ (%)
	T_deg onset_^(1)^ (°C)	T_max_^(2)^ (°C)	Mass Loss (%)		
WS	231.0	364.8	65.4	258.3	25.0
PsS	257.4	357.7	70.8	275.4	22.4
AS	220.7	371.1	74.8	270.6	18.8
DS	223.6	319.3	71.1	261.9	22.6
PS	270.3	373.1	68.1	284.7	23.0

^(1)^ The onset of degradation temperatures evaluated with the software TRIOS 5.0 (TA Instruments, New Castle, DE, USA). ^(2)^ The temperature associated with the highest rate of the main degradation step (DTGA peak). ^(3)^ The temperature associated with 10% of the mass loss. ^(4)^ The residual mass at 650 °C.

## Data Availability

Not applicable.
